# Angiopoietins stimulate pancreatic islet development from stem cells

**DOI:** 10.1038/s41598-021-92922-5

**Published:** 2021-06-30

**Authors:** Soujanya S. Karanth, Shuofei Sun, Huanjing Bi, Kaiming Ye, Sha Jin

**Affiliations:** 1 Department of Biomedical Engineering, Binghamton University, State University of New York (SUNY), Binghamton, NY 13902 USA; 2 Center of Biomanufacturing for Regenerative Medicine, Binghamton University, State University of New York (SUNY), Binghamton, NY 13902 USA

**Keywords:** Cell biology, Induced pluripotent stem cells

## Abstract

In vitro differentiation of human induced pluripotent stem cells (iPSCs) into functional islets holds immense potential to create an unlimited source of islets for diabetes research and treatment. A continuous challenge in this field is to generate glucose-responsive mature islets. We herein report a previously undiscovered angiopoietin signal for in vitro islet development. We revealed, for the first time, that angiopoietins, including angiopoietin-1 (Ang1) and angiopoietin-2 (Ang2) permit the generation of islets from iPSCs with elevated glucose responsiveness, a hallmark of mature islets. Angiopoietin-stimulated islets exhibited glucose synchronized calcium ion influx in repetitive glucose challenges. Moreover, Ang2 augmented the expression of all islet hormones, including insulin, glucagon, somatostatin, and pancreatic polypeptide; and β cell transcription factors, including NKX6.1, MAFA, UCN3, and PDX1. Furthermore, we showed that the Ang2 stimulated islets were able to regulate insulin exocytosis through actin-filament polymerization and depolymerization upon glucose challenge, presumably through the CDC42-RAC1-gelsolin mediated insulin secretion signaling pathway. We also discovered the formation of endothelium within the islets under Ang2 stimulation. These results strongly suggest that angiopoietin acts as a signaling molecule to endorse in vitro islet development from iPSCs.

## Introduction

Diabetes is a global epidemic posing significant challenges for human health and wellbeing. While islet transplantation is the most promising treatment and is seen as a ‘cure’ to diabetes^1^, the major hindrance is the scarcity of donor islets. Stem cells can be an unlimited source for generating islets. In particular, islets derived from human induced pluripotent cells (iPSCs) have an added advantage of the reduced risk of graft rejection, as these cells could be derived from the patient’s own cells^2^. These concepts have evolved with promising, positive and steady progress in the past decades. Earlier studies from our and other groups have showed the possibility of generating glucose-responsive insulin-secreting cells from iPSCs by recapitulating the in vivo developmental processes using a stepwise differentiation protocol^3–13^. More recently, efforts have been made for generating islet-like organoids, consisting of major islet hormone-secreting cell subsets, including α, β, δ, and pancreatic polypeptide (PP) from stem cells^11–13^.

Mounting evidence suggests that β cell alone may not offer a long-term solution to maintaining normoglycemia in diabetic patients, as both type 1 (T1D) and 2 diabetes (T2D) are “bi-hormonal disorder” diseases^14^. α cell dysfunction is also a major cause of excessive hepatic glucose production and insulin resistance in both T1D and T2D patients^15–17^. In addition, the heterotypic interactions between α and β cells are pivotal to maintaining glucose homeostasis in the blood^18–21^. Somatostatin secreted from δ cells is crucial to balancing endocrine hormone production^22^. While PP cells are one of the most poorly understood cell types in islets, pancreatic polypeptide (PPY) secreted from PP cells is found to be correlated to somatostatin levels in response to eating and fasting^23,24^. These evidences strongly suggest the necessity of generating whole islets that encompass not only β, but also α, δ, and PP cells. This necessity becomes more profound when human islets are required for drug discovery and diabetes pathophysiological studies.

However, the generation of physiologically functional islets has had limited success, due in part to our poor understanding of tissue niches essential to islet assembly and development in vitro. Particularly, our knowledge of acquired microenvironments indispensable for multicellular islet differentiation and maturation is lacking. Hence, the directed iPSC islet development must be improved in order to realize robust production of islets from iPSCs for diabetes treatment, pathophysiological study, and drug discovery.

We have previously reported that decellularized pancreatic extracellular matrix (dpECM) contains instructive molecules, such as type V collagen, that endorse islet development from iPSCs^11,13^. Our proteomic analysis of dpECM revealed the presence of another signaling molecule, i.e. angiopoietin-2 (Ang2). Ang2 is an endothelial growth factor. It is also a modulator of endothelial permeability^25^. It has been reported that Ang2 contributes to endothelial cell (EC) activation, the initiation of angiogenesis, and pancreatic vascularization^25,26^. In vivo, Ang2 is exclusively secreted from ECs and is stored in ECs’ Weibel–Palade bodies (small storage granules)^27^. The expression of Ang2 is rapidly induced in stressed ECs to mitigate apoptosis^28^. Accumulating evidence shows an intimate interplay between the endothelium and endocrine cells of the pancreas, which influences the development, function, and survival of the pancreas^29–33^. ECs secrete cytokines, such as HGF, CTGF, and thrombospondin-1 to orchestrate organogenesis, although their complex and dynamic interactions are largely unknown^34–36^. While angiopoietins have been reported principally for their role in angiogenesis^37,38^ and non-vascular system^39–41^, to the best of our knowledge, the cellular actions of Ang2 on pancreatic islet development from stem cells have not been reported or explored.

In this study, we investigated whether angiopoietin can act as a singling molecule to augment the maturation of islets during iPSC islet development. We discovered that the exposure of pancreatic progenitors to either angiopoietin-1 (Ang1) or Ang2 at late-stages of iPSC islet development led to the development of physiologically functional islets. These organoids were capable of regulating insulin exocytosis through dynamic polymerization and depolymerization of actin-filament (F-actin) upon glucose challenge. In addition, the angiopoietin signal elevated the glucose synchronized calcium ion (Ca^2+^) influx of iPSC-derived islet cells in repetitive glucose challenges. Likewise, it enhanced the expression of not only all islet hormones, including insulin, glucagon, somatostatin, and pancreatic polypeptide; but also β cell transcription factors, including NKX6.1, MAFA, UCN3, and PDX1. Mechanistic study indicated that Ang2 stimulated islets were able to regulate insulin exocytosis by activating the CDC42-RAC1-gelsolin-mediated insulin secretion pathway. Furthermore, Ang2 induced the formation of intra-islet endothelium. These results demonstrated, for the first time, a unique role that  angiopoietin plays in the iPSC islet development.

## Results

### Ang2 promotes in vitro islet development

**Figure 1:**
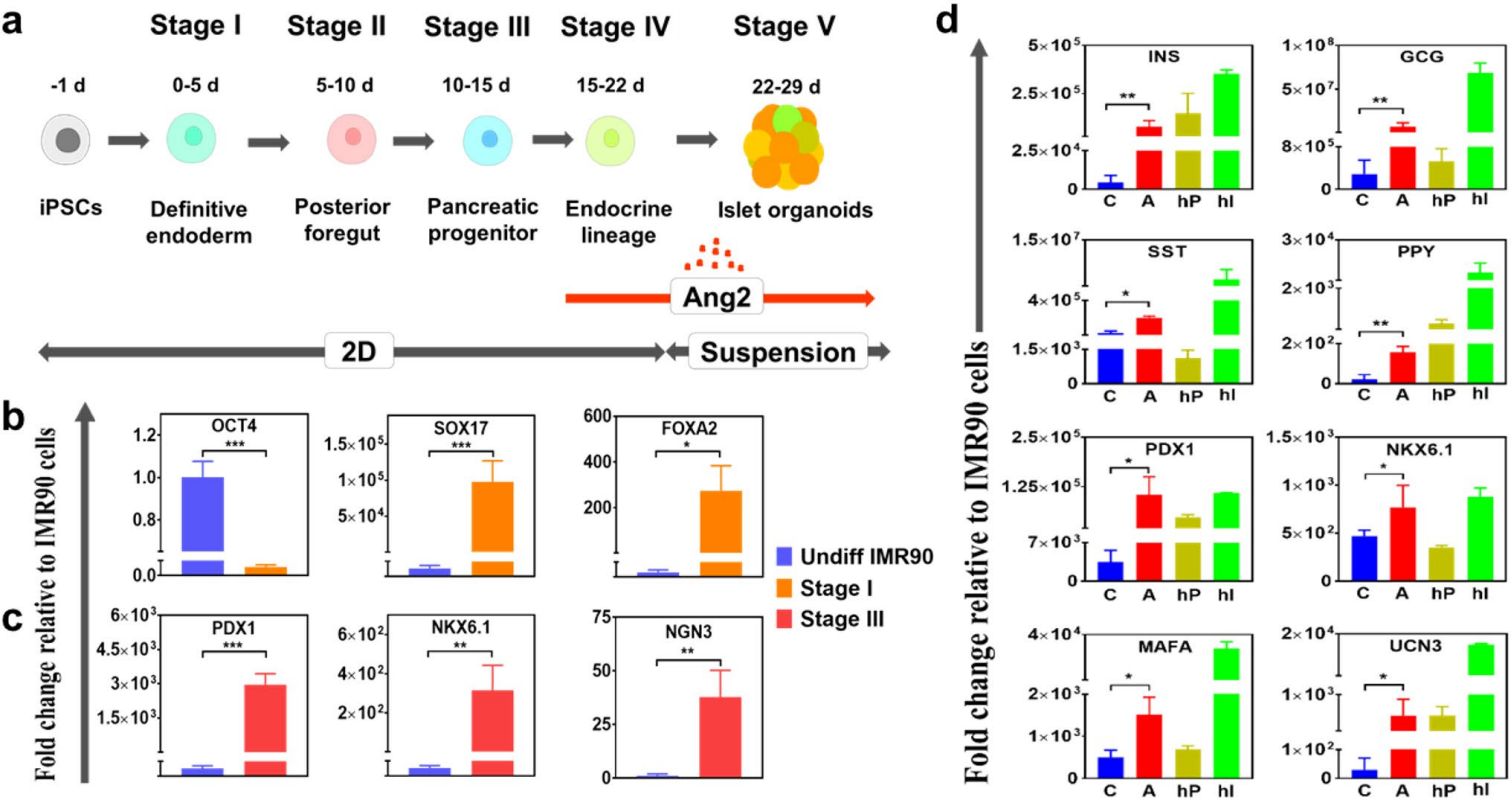
Outline of the stepwise differentiation procedure and key signature marker gene expressions during iPSC islet development. (**a**) A schematic diagram of a five-stage islet development protocol. (**b**,**c**, **d**) iPSC-derived cells were characterized for their pancreatic marker gene expressions at stage of definitive endoderm (**b**), pancreatic progenitor (**c**), and islet organoid (**d**). The expression levels were normalized to IMR90 cells. C: control; A: Ang2. Human pancreas RNA (hP) and human islet RNA (hI) were used as positive controls. Results were obtained from four independent differentiation experiments and shown as mean ± SD. *p < 0.05, **p < 0.01, and ***p < 0.001.

A five-stage differentiation protocol developed in our previous study was adopted with modification for this study^11^. As illustrated in Fig. 1a, Stage I generates definitive endoderm (DE) cells, whereas Stage II and Stage III produce pancreatic progenitors committed to endocrine lineage. Stage IV generates four major islet cell subsets, including α, β, δ, and PP cells. Stage V allows the formation of islet cell clusters that mature into islets. Ang2 was added to the differentiation medium from Stage IV to Stage V. The timing of addition was chosen based on our pre-test. The islet cells were characterized by quantitative real-time PCR (qRT-PCR) at the early, intermediate, and end stages of the islet development (Fig. 1b–d). We observed substantially elevated expressions of DE marker genes SOX17 and FOXA2 (Fig. 1b), along with the loss of pluripotent gene marker OCT4 at Stage I. The expression levels of pancreatic progenitor marker genes PDX1, NKX6.1, and NGN3 were significantly high at Stage III (Fig. 1c). The expression levels of all islet hormone markers, including insulin (INS), glucagon (GCG), somatostatin (SST), and pancreatic polypeptide (PPY) increased considerably at Stage V under Ang2 stimulation (Fig. 1d). In addition, the expressions of mature β cell transcription factors NKX6.1, MAFA, UCN3, and PDX1 elevated remarkably under the Ang2 stimulation (Fig. 1d), signifying that Ang2 may augment islet maturation during iPSC islet development.

**Figure 2:**
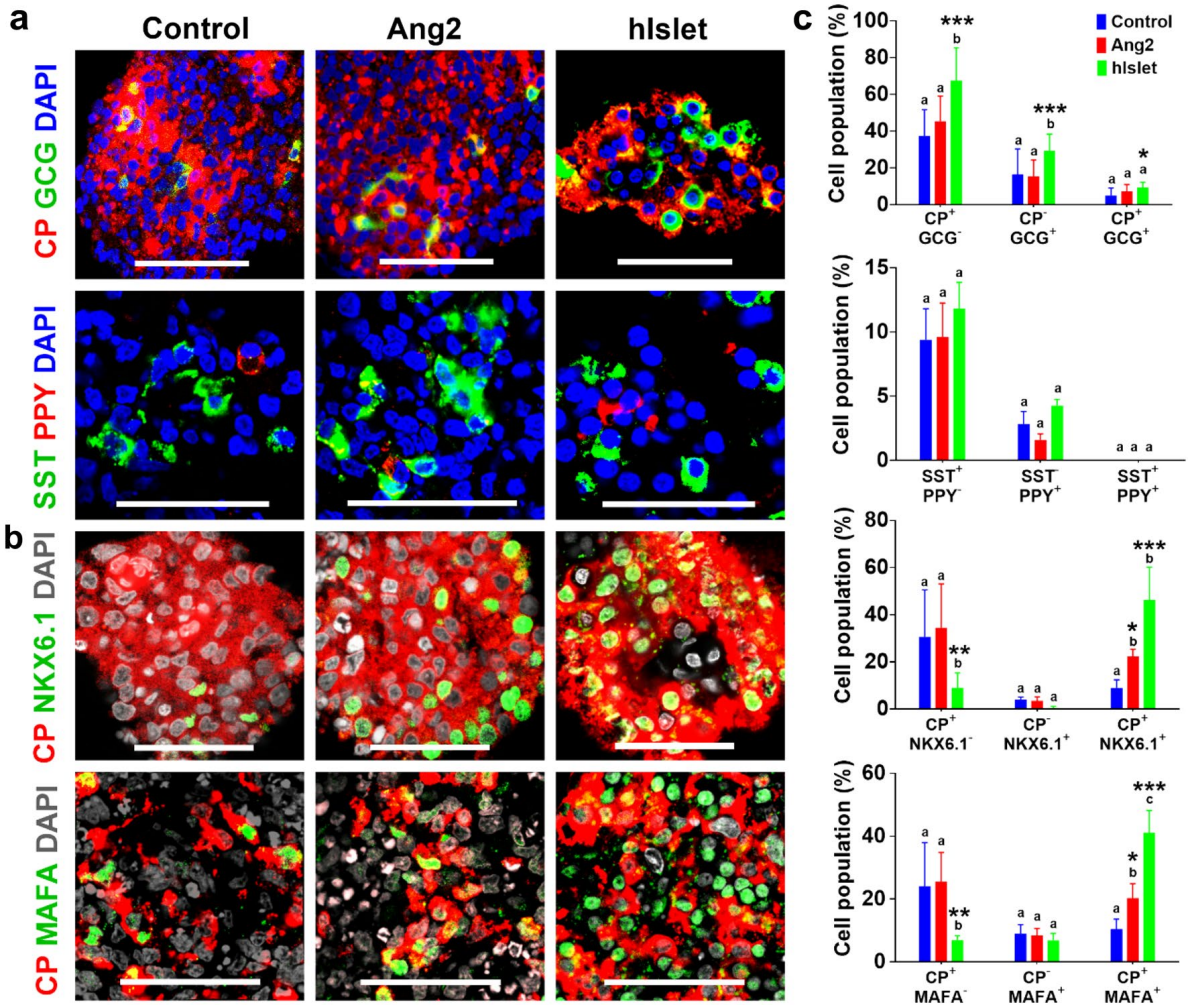
Representative organogenesis of iPSC-derived islet organoids. At the end of differentiation, the islets were immunofluorescently labeled for (**a**) C-peptide (CP, red) and glucagon (GCG, green), somatostatin (SST, green) and pancreatic polypeptide (PPY, red). (**b**) NKX6.1 (green) and CP (red), and MAFA (green) and CP (red). Cells were counterstained with DAPI (grey). Scale bars, 50 μm. Human islets (hIslet) served as a positive control. (**c**) Semi-quantitative analysis of cellularity of the islets. Image analysis was performed using ImageJ software (n = 7–16 images for each condition). Results are shown as mean ± SD. Different letters indicate significant differences between the groups and *p*-value represented as *p < 0.05; **p < 0.01; and ***p < 0.001 compared to the control group.

Next, we examined the phenotype and tissue architecture of the islets by immunofluorescence staining of hormone-secreting islet cell subsets. As shown in Fig. 2a, we detected C-peptide (CP)^+^ β, GCG^+^ α, SST^+^ δ, and PPY^+^ PP subsets in the islet organoids. Further analysis of C-peptide producing cells in the islets revealed the localization of mature β cell transcription factors, NKX6.1 and MAFA, inside cell nucleus (Fig. 2b). To determine the cellularity of the islets, we analyzed the images (n = 7–16 at each experimental condition) using ImageJ software (Fig. 2c). We found that subsets of hormone-producing cells in the control and Ang2 groups were similar. Islets formed in the presence or absence of Ang2 all consisted of CP^+^/GCG^−^, CP^−^/GCG^+^, SST^+^/PPY^−^ and SST^−^/PPY^+^. Remarkably, the percentages of CP^+^/NKX6.1^+^ and CP^+^/MAFA^+^ cells increased considerably in the Ang2 group. The islets in the Ang2 group contained 20.4 ± 5.7% C-peptide (CP)^+^/NKX6.1^+^ cells, 18.2 ± 6.9% CP^+^/MAFA^+^, whereas the control group encompassed only 8.8 ± 3.7% CP^+^/NKX6.1^+^ cells and 11.3 ± 2.4% cells CP^+^/MAFA^+^. Earlier studies by other groups have shown that cells co-expressing CP and NKX6.1 are mature β cells that possess a better glucose responsive ability^42,43^. These results revealed that β cells generated in the Ang2 group were more mature than those in the control group.

### Angiopoietins permit iPSC-derived islet cells to release insulin in a glucose-responsive manner

**Figure 3:**
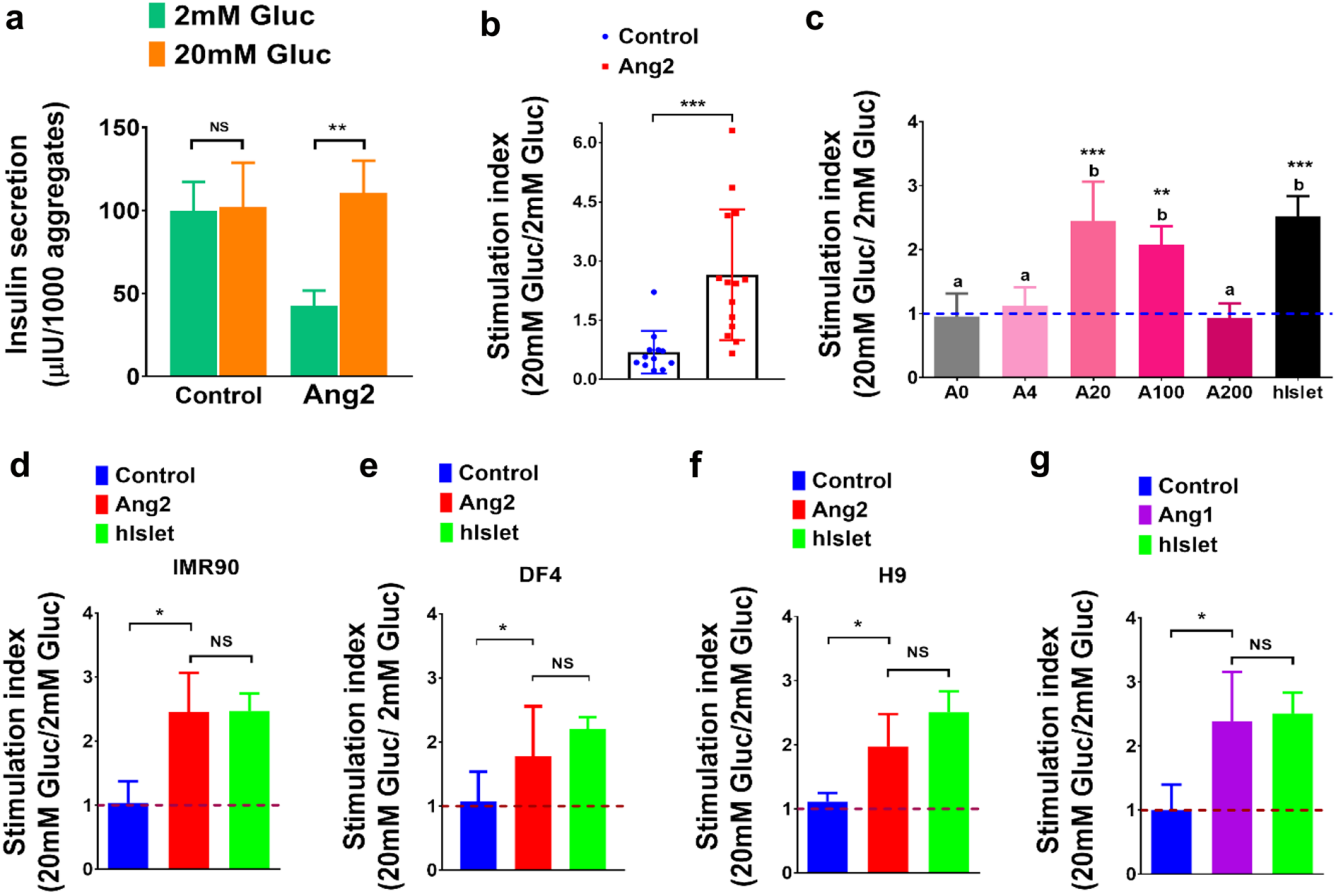
Angiopoietins promote the development of islets with elevated glucose-responsive insulin secretion capacity from differentiation of multiple pluripotent stem cell lines. (**a**) Parallel glucose-stimulated insulin secretion from islets generated in the absence or presence of Ang2 (n = 6). (**b**) Insulin stimulation index from consecutive three rounds of low (2 mM)–high (20 mM) glucose challenge measured from iPSC-derived islets by sequential glucose-stimulated insulin secretion analysis. Control (n = 12) and Ang2 (n = 14). (**c**) Stimulation index of insulin secretion from iPSC-derived islets in the presence of Ang2 at 0 (A0), 4 (A4), 20 (A20), 100 (A100) and 200 ng/ml (A200) (n = 4). Groups with different letters (a or b) represent significant differences. (**d-f**) Insulin stimulation index of islet organoids generated from iPSC line IMR90 (n = 4) (**d**), iPSC line DF4 (n = 6) (**e**), and hESC line H9 (n = 3) (**f**), respectively. (**g**) Insulin stimulation index of iPSC-derived islets generated in the presence of Ang1 (n = 4). All the results are shown as mean ± SD. *p < 0.05, **p < 0.01, ***p < 0.00, and NS: not significant.

We next conducted a static parallel glucose-stimulated insulin secretion (p-GSIS) assay to ascertain the function of Ang2 stimulated islets (Fig. 3a). Islets generated in the absence of Ang2 served as a control. The control group exhibited a non-regulated insulin secretion. These cells released similar amounts of insulin when being challenged with either low (2 mM) or high glucose (20 mM). The stimulation index, which denotes the ratio of insulin secretion at high (20 mM) to low glucose (2 mM), was 1.0 ± 0.3 (Fig. 3b). Interestingly, the Ang2 group displayed an increased insulin release under a high glucose concentration and a decreased-basal level insulin release under a low glucose concentration. The insulin stimulation index was 2.5 ± 0.5-fold, equivalent to that observed from human islets 2.5 ± 0.3 (Fig. 3a–c). Furthermore, the stimulation index was examined by three rounds of serial challenges of 2 mM and 20 mM glucose. The control group failed to distinguish between a minor and a major increase in glucose, while the Ang2 group retained glucose responsiveness for all rounds of sequential low–high glucose challenges, as quantified by the stimulation index (Fig. 3a,b). It is worth noting that we did not use human donor islets to perform sequential GSIS (s-GSIS), due to the fact that other groups had shown that the stimulation index of human islets is maintained at 2.0 most of the time, although there is a high variability in an individual donor’s absolute magnitude of insulin secretion^9^. We did examine the stimulation index of human donor islets repeatedly. The results from these tests confirmed that the insulin stimulation index is maintained at 2.0 in all the tests we performed (data no shown).

To determine whether the effect of Ang2 on islet development is dose-dependent, we differentiated iPSCs at varied concentrations of Ang2 from 0, 4, 20, 100, to 200 ng/ml. As shown in Fig. 3c, islet cells generated in the presence of Ang2 at 20 and 100 ng/ml showed a stimulation index of 2.5 ± 0.6 and 2.1 ± 0.3, respectively, which is equivalent to human donor islets 2.5 ± 0.3. The islets generated at 4 and 200 ng/ml of Ang2 showed no difference as compared to the control group. Hence, we confirmed that the effect of Ang2 on islet development is dose-dependent. To ensure rigor of the study, we determined the effect of Ang2 on islet development using another iPSC line DF4 and human embryonic stem cell (hESC) line H9. Different from IMR90 that was prepared from a female donor, DF4 was derived from a male donor. We differentiated both DF4 and H9 into islets under the exactly same conditions shown in Fig. 1a. As shown in Fig. 3e,f, the insulin stimulation index of the islets formed from DF4 and H9 in the presence of 20 ng/ml of Ang2 was 1.8 ± 0.7 and 1.9 ± 0.4, respectively, which is comparable with that of IMR90-derived islets (Fig. 3d), suggesting that the effect of Ang2 on islet development is not cell line-specific.

Ang1 is another major protein in the angiopoietin family. It shares 60% amino acid homology with Ang2^44^. Therefore, we investigated whether Ang1 also plays a similar role in the islet development as Ang2. Remarkably, the islets developed in the presence of Ang1 (20 ng/ml) exhibited an insulin stimulation index of 2.4 ± 0.7, which is similar to that detected from Ang2 induced islets (Fig. 3g). Hence, similar to Ang2, Ang1 can also induce the development of glucose-responsive islets. These experimental results clearly demonstrated the augmentation of iPSC islet development by angiopoietin factors.

It should be noted that there are no reliable markers to sort islets from non-islet cell clusters^11^. We found no significant difference in the total amount of insulin released from the control and Ang2 groups at high glucose level (Fig. 3a). Therefore, we did not focus on quantifying islet cells by flow cytometry; rather, we explicitly focused on assessing glucose-responsive insulin secretion from these islets, as described below. We sought to specifically examine the improved glucose responsiveness of the islets generated under the Ang2 stimulation. To this end, we extended our study to a rat β cell line RIN-5F to examine whether Ang2 signaling can augment these cells’ glucose sensitivity. RIN-5F line does not possess glucose sensitivity. It was reported that these cells secrete insulin in a non-glucose responsive fashion^45^. We cultured the cells in the presence of Ang2 from 0, 20, and 100 ng/ml for 3 days and then performed GSIS assay (Fig. S1). Ang2 stimulation failed to improve these cells’ glucose sensitivity, as they released the same amount of insulin regardless of glucose level change, which coincides with the previous report^45^. We speculated that Ang2 stimulation affects the maturation of β cells, but has no effect on the glucose-responsive insulin secretion capability of the terminally differentiated β cells. To further confirm these observations, we characterized glucose synchronous Ca^2+^ influx in Ang2 stimulated islets.

### Angiopoietins regulate dynamics of calcium ion influx in response to different glucose levels

**Figure 4:**
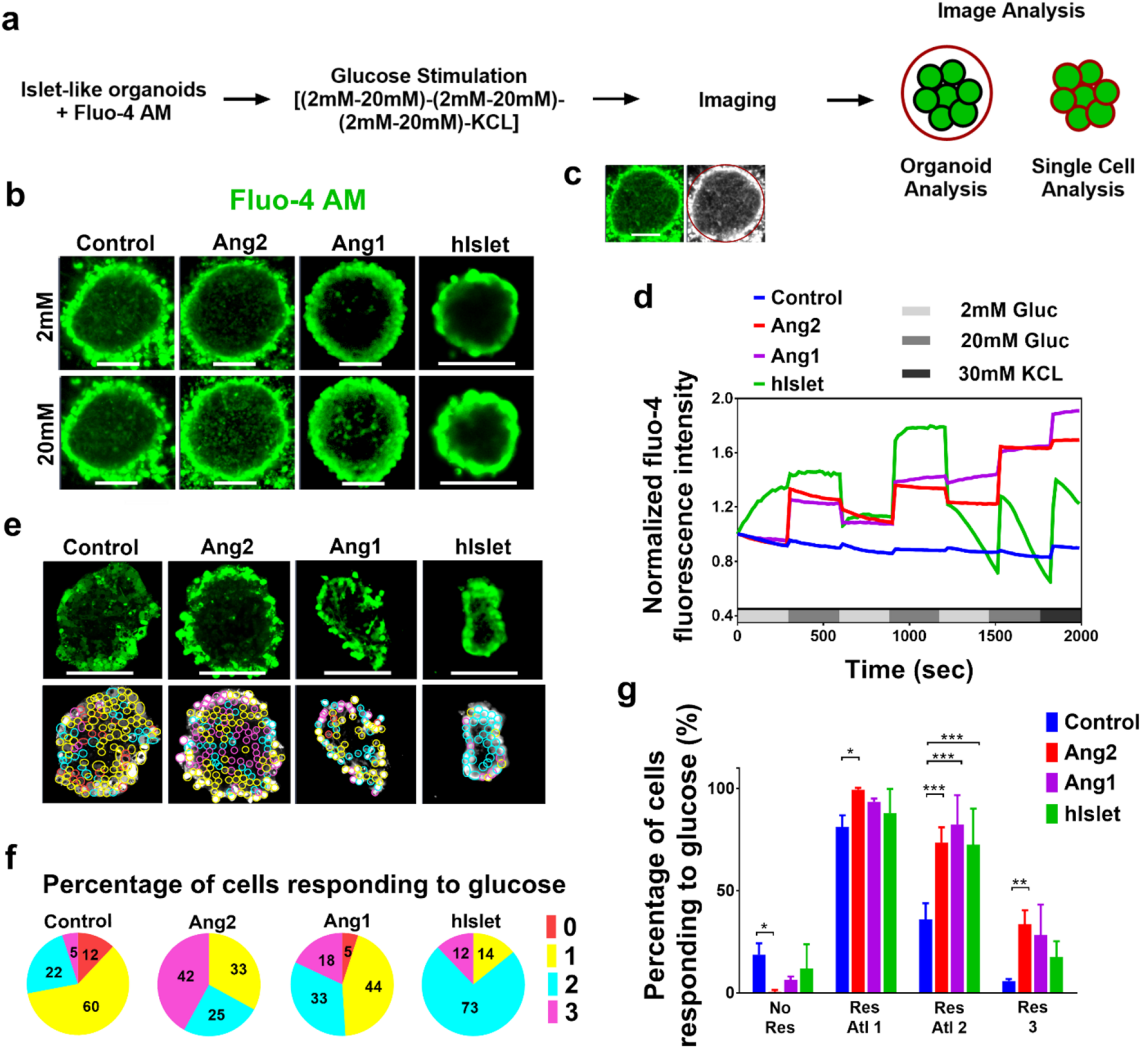
Angiopoietins facilitate the development of islets with enhanced glucose-responsive cellular Ca^2+^ influx in insulin-secreting cells. (**a**) A regimen for imaging cellular Ca^2+^ influx. (**b**) Representative micrographs of the iPSC-derived islets stained with Fluo-4 upon low (2 mM) or high (20 mM) glucose challenge. Scale bars: 100 μm. (**c**) Imaging analysis of Ca^2+^ dynamics for a subset of cell populations within an islet organoid. (**d**) Ca^2+^ dynamics behavior depicted as average of the Fluo-4 fluorescence intensity obtained from each experimental group. These values were from a population of cells within the islets (n = 9) and donor islets (n = 4) over the entire course of sequential glucose stimulations. (**e**) Fluo-4 stained insulin-secreting cells responding to sequential low–high glucose stimulations all three, two, and one times were circled with magenta, blue, and yellow respectively. Red circles denote cells that did not respond to any glucose challenge. Scale bar: 100 μm. (**f**) Percentage of cells in the control, Ang2, and Ang1 groups, whose Ca^2+^ influx responded to sequential (low–high) glucose changes. Human islets (hIslet) served as a positive control. Red: nonresponding cells; yellow, blue, and magenta: cells’ Ca^2+^ influx responded to glucose change once, twice, and thrice. More than 400 cells were counted in each islet organoid. All of the experiments were performed in triplicate. (**g**) The cells’ Ca^2+^ influx changes responding to sequential low–high glucose level challenge. Three islets with single cell number n > 400 and human islets with cell number n > 250 were analyzed. No Res, Res Atl 1, Res Atl 2, and Res 3 denote to cells’ Ca^2+^ influx responded to glucose level changes zero time, at least once, at least twice, and all three times, respectively. *p < 0.05, **p < 0.01, and ***p < 0.001.

The Ca^2+^ influx is intimately associated with glucose-stimulated insulin exocytosis in β cells^46,47^. An increase in blood glucose levels causes Ca^2+^ influx in β cells, which in turn leads to insulin exocytosis. To scrutinize whether the glucose-responsive insulin secretion from the islets generated under the angiopoietin stimulation represents glucose level regulated Ca^2+^ influx, we performed semi-quantitative time series imaging analysis of Fluo-4 stained single β cells in the islets (Fig. 4a–c). Fluo-4 has been widely used as a Ca^2+^ indicator^48^. The representative Ca^2+^ influx in the control, Ang2, Ang1 groups, and human islets (hIslet) are shown in Fig. 4b. The Ca^2+^ influx in the control group remained constant with no rise upon high glucose challenge (Fig. 4d). In contrast, the Ca^2+^ influx in the Ang1 and Ang2 groups rose upon high glucose challenge. It dropped to a basal level at a low glucose level. This Ca^2+^ influx was similar to that observed in human islets. The area under the curve (a.u.c) for Ca^2+^ influx response at all low glucose levels was compared to the Ca^2+^ influx response curves detected upon high glucose challenges. The a.u.c of Ca^2+^ influx response under high glucose stimulations in the Ang1 and Ang2 groups was significantly greater than that at low glucose levels (Fig. 4d). After the three rounds of consecutive low–high glucose challenges, the islets were treated by KCl for islet depolarization. The human islets showed immediate response to KCl treatment in the form of a sharp increase in Ca^2+^ influx followed by a rapid drop. This might attribute to deterioration of these islets during donor isolation procedure, preservation, shipping, and repeated treatment in an imaging chamber. iPSC-derived islets in the Ang1 and Ang2 groups exhibited a certain degree of increase in Ca^2+^ influx after KCl challenge. The results suggested that these islets need to be matured further.

We next carried out Ca^2+^ influx analysis of single cells inside the islets. The single cells that responded to cyclic low (2 mM) and high (20 mM) glucose challenges once (1), twice (2), and thrice (3) were distinguished as shown in Fig. 4e. In the control group, approximately 5% of cells responded all three times, 22% of cells responded twice, 60% of cells responded once, and 12% of cells did not respond to glucose level change anytime (Fig. 4f). In contrast, 42% of cells responded thrice, 25% of cells responded twice, and 33% of cells responded once in the Ang2 group. In the Ang1 group, 18% of cells responded thrice to glucose level changes, 33% of cells responded twice, and 44% of cells responded once. As a positive control, 12% of human adult islet cells responded all three times, 73% of cells responded twice, and 14% of cells responded once. When the analysis was extended to multiple islet organoids in each group (Fig. 4g), the cells responding to every glucose challenge (Fig. 4g Res 3) were the highest in the Ang2 group with a percentage of 33.8 ± 6.6 cells. In the Ang2 group, the percentage of cells responding at least once (Res Atl 1) was 99.45 ± 1.0 and the percentage of cells responding at least twice was 73.58 ± 7.6. Collectively, the percentage of glucose-responsive cells generated under either Ang1 or Ang2 stimulation was significantly higher than the control group as indicated by all the responding frequencies of Ca^2+^ influx measured. In addition, the percentages of glucose-non-responsive cells (No Res) in the Ang1 and Ang2 groups were significantly lower than that in the control group.

### Ang2 facilitates F-actin remodeling in response to glucose level changes

**Figure 5:**
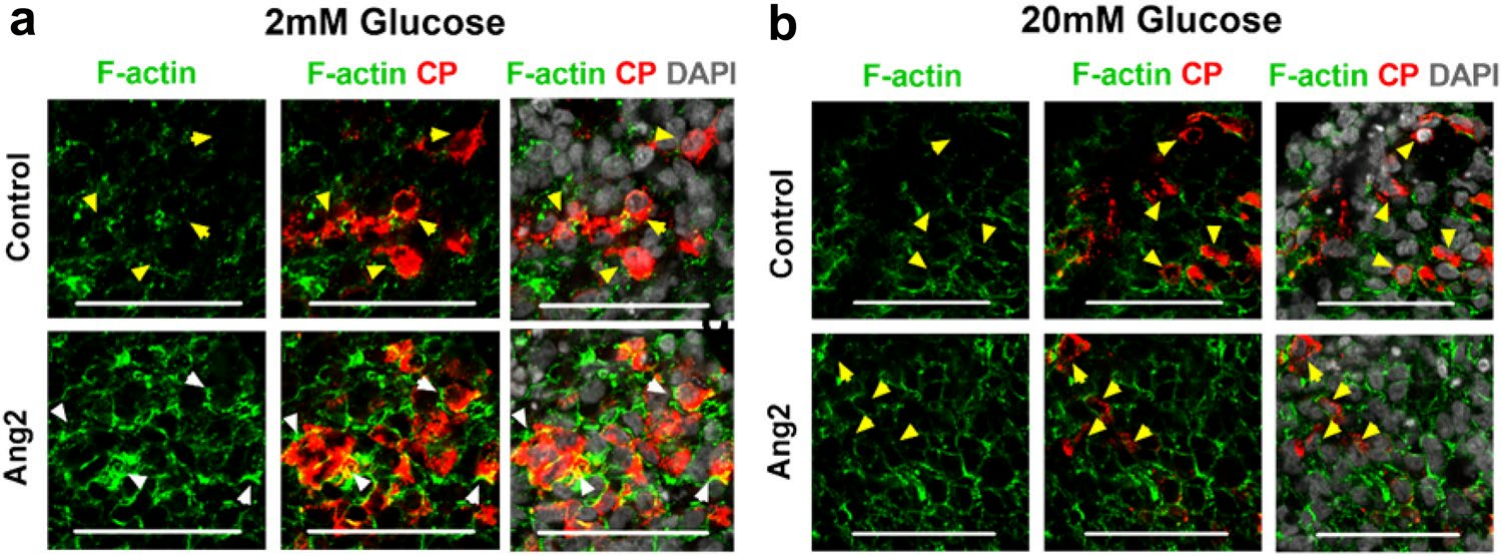
Ang2 regulates glucose-responsive insulin secretion through dynamic F-actin remodeling. Representative micrographs of F-actin organization in insulin-secreting cells at (**a**) 2 mM and (**b**) 20 mM glucose challenge. White arrowheads indicate thick and highly polymerized F-actin. Yellow arrowheads indicate depolymerized F-actin**.** Scale bars: 50 µm.

It has been reported that F-actin of β cells is involved in insulin granule mobilization and cell membrane fusion to facilitate insulin exocytosis^49^. We determined whether Ang2 facilitates F-actin rearrangement during insulin exocytosis upon glucose challenge. We observed that the F-actin polymerized into a thick barrier around the Ang2 stimulated β cells, restricting the insulin within the cells under low glucose condition (Fig. 5a). Under a high glucose condition, the F-actin depolymerized into discontinuous thinner fragments, facilitating the insulin release from these cells (Fig. 5b). Conversely, no such F-actin rearrangements were observed in the control group. The F-actin remained depolymerized under both low and high glucose conditions.

**Figure 6:**
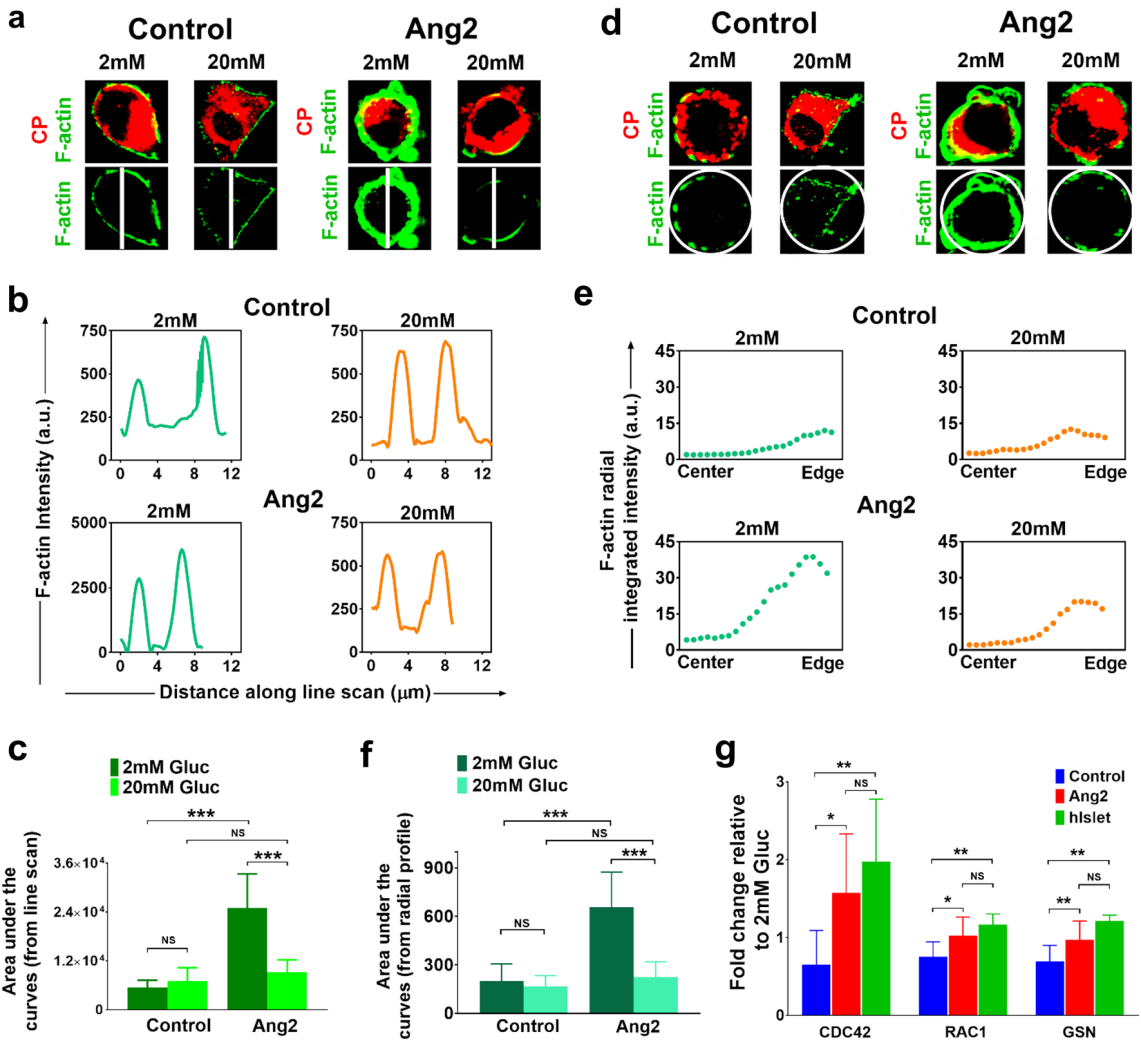
Characterization of F-actin patterning and key molecules involved in glucose-responsive insulin exocytosis. (**a**) Line scanning analysis of individual insulin secreting cells upon 2 mM or 20 mM glucose challenge (n = 15–30 cells at each condition). (**b**) F-actin intensity patterns across the individual insulin-secreting cell upon low or high glucose challenge. (**c**) Average F-actin intensity determined by line scanning of individual insulin-producing cells upon low or high glucose challenge (n = 15–30 cells at each condition). (**d**) Representative images of individual C-peptide producing cells analyzed in control and Ang2 groups upon 2 mM or 20 mM glucose challenge (n = 15–30 cells at each condition). (**e**) Representative curves of the integrated F-actin intensity from the center to edge of a β cell in the control and Ang2 groups. F-actin intensity in all 360 °C directions was characterized along the radius to obtain radial mean intensity. The x-axis represents distance from center to the edge of a β cell. (**f**) Average F-actin intensity at low and high glucose conditions determined by radial profiling (n = 15–30 cells at each condition). (**g**) Relative gene expressions of key molecules involved in glucose-responsive insulin exocytosis. iPSC-derived islets generated in the presence or absence of Ang2 stimulation at Stage V were challenged with low (2 mM) or high (20 mM) glucose, followed by RNA extraction and qRT-PCR analysis. The ratio of gene expression at high to low glucose was assessed. Human donor islets (hIslet) served as controls. Results are shown as mean ± SD; *p < 0.05, **p < 0.01, ***p < 0.001, NS: non-significant.

To quantitatively assess the F-actin remodeling, we performed a systematic characterization of the F-actin expression of individual β cells. We observed that the F-actin intensity at the cell edges of the control group remained around 200–750 arbitrary units (a.u) under both low and high glucose levels (Fig. 6). Remarkably, the F-actin intensity at the cell edges of the Ang2 group reached to 2500–3750 a.u. under a low glucose condition, but dropped to 500 a.u upon a high glucose challenge (Fig. 6b). The overall F-actin intensities under low and high glucose challenges in both control and Ang2 groups were compared by analyzing area under the F-actin intensities using approximately 15–30 β cells from each group (Fig. 6c). F-actin intensity under low glucose was significantly higher than that under high glucose in the Ang2 group. In contrast, the F-actin intensity of the control group under low glucose was significantly lower as compared to that of the Ang2 group, suggesting defective glucose responsive F-actin rearrangement in islet cells generated in the absence of Ang2. Accordingly, Ang2 facilitates the dynamic regulation of F-actin remodeling of insulin-producing cells for physiological insulin release.

Furthermore, we implemented radial profiling of F-actin intensity to validate the line scanning analysis using ImageJ software (Fig. 6d–f). The F-actin intensity was evaluated from 360 °C of encircling the insulin-producing cells (Fig. 6d). This yielded F-actin expression curves of mean intensity along the radius, i.e. from center to the edge of the β cells (Fig. 6e). The curves generated consisted of prominent peaks at the edge. At low glucose condition, a higher F-actin intensity peak reflecting highly polymerized F-actin was observed towards the edge in the Ang2 group, as compared to that of the control group (Fig. 6e). The circularly analytical results are consistent with the line scanning analysis. Moreover, no difference was observed in the F-actin intensity between the control and Ang2 groups at high glucose condition (Fig. 6).

It has been recognized that mature β cells utilize cell division control protein 42 (CDC42) and Ras-related C3 botulinum toxin substrate 1 (RAC1) to mobilize and dock insulin granules to the plasma membrane^50,51^. Gelsolin (GSN) has been reported to facilitate F-actin reorganization by mediating glucose-dependent actin depolymerization to release insulin from the cells^52,53^. Hence, we investigated whether Ang2 stimulated islet cells utilize these molecules to regulate glucose-responsive insulin release. We collected the islets at the end of the development and challenged them with low (2 mM) or high (20 mM) glucose, followed by assessing the expression of CDC42 and RAC1 at high or low glucose. As shown in Fig. 6g, the ratio of CDC42 expressed at high to low glucose increased approximately 2.4-fold in the Ang2 group, as compared to that in the control group. There was no significant difference between the Ang2 and the human donor islet groups (Fig. 6g). RAC1 and GSN in the Ang2 group were also slightly increased as compared to that in the control group, and their sensitivity to glucose was similar to that of the human islets (Fig. 6g). These results suggested that Ang2 augmented the islets’ glucose-responsiveness by enhancing their insulin secretion pathway for insulin release.

### Ang2 induces endothelium formation during iPSC islet development

**Figure 7:**
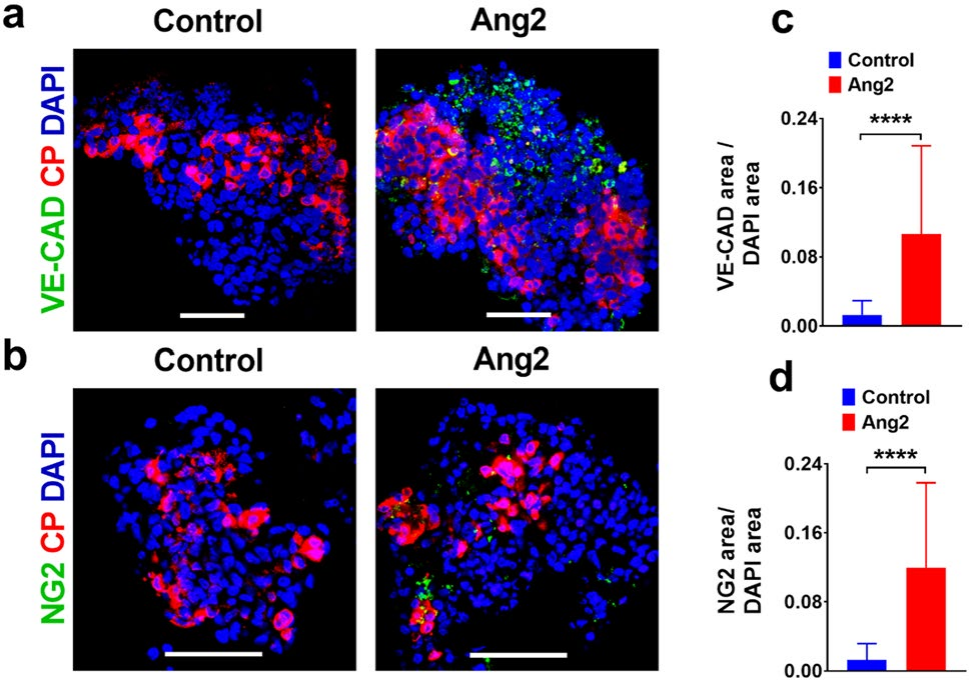
Ang2 induces the generation of endothelial cells and pericytes in iPSC derived islet microenvironment. (**a**) Representative images of VE-cadherin and C-peptide immunofluorescence co-staining in islet-like organoids. (**b**) Representative images of NG2 and C-peptide immunofluorescence co-staining in islet-like organoids. (**c**,**d**) The ratio of VE-Cadherin^+^ endothelial area/DAPI area and NG2^+^ pericyte area/DAPI area in islet-like organoids. Scale bars: 50 µm. n = 19–35 islet organoids for each condition. Results are shown as mean ± SD. ****p < 0.0001.

Ang2 is known to initiate angiogenesis and facilitate vascular permeability^54,55^. Pericytes interact with ECs in the islet endothelium environments^56^. To gain insights into the interplays between endothelium and islet organogenesis, we interrogated whether Ang2 induces angiogenesis during iPSC islet development, leading to the production of mature islets. We examined the expression of an endothelial marker, vascular endothelial cadherin (VE-cadherin) and a pericyte marker NG2 in islets at Stage V by immunofluorescence microscopy. As expected, we detected a wide spread of VE-cadherin^+^ ECs and NG2^+^ pericytes within Ang2 stimulated islets (Fig. 7a,b), suggesting the formation of intra-islet endothelium. Further analysis showed that the ratio of VE-cadherin area/DAPI area and the ratio of NG2 area/DAPI area were significantly higher in the Ang2 group (Fig. 7c,d). These results indicated that Ang2 induces initiation of new endothelial microenvironment within islet organoids.

## Discussion

In this study, we report, for the first time, an inductive effect of angiopoietins on iPSC islet development and maturation. We demonstrated that Ang2 induced islet maturation and the formation of intra-islet endothelium. Mechanistic study revealed active F-actin remodeling aided by activated CDC42-RAC1-gelsolin pathway in addition to glucose level synchronous Ca^2+^ influx dynamics in Ang2 stimulated islets. By applying angiopoietin factors such as Ang1 or Ang2 at late-stages of islet development, we discovered that glucose sensitivity of the angiopoietin stimulated islets increased significantly. We showed that the islets generated without angiopoietin stimulation did not possess glucose-responsive insulin secretion capability. They secreted the same amount of insulin all the time even at a low glucose condition, indicating their immaturity and failure to release insulin in a physiological fashion. In contrast, the islets developed under angiopoietin stimulation were able to secrete insulin in a glucose-responsive manner. Through analysis of F-actin remodeling, a critical step of glucose-responsive insulin-secretion in mature islet cells, we found that insulin-secreting cells generated in the presence of angiopoietin signaling were able to repattern F-actin in response to glucose level changes to facilitate glucose-responsive insulin-release. As shown in Fig. 6a,d, insulin was constrained inside the cell cytoplasm by a thicker layer of F-actin surrounding the cell membrane of islets generated under Ang2 stimulation upon low glucose challenge. The F-actin layer was dispersed to enable the secretion of insulin upon high glucose challenge. This F-actin depolymerization is one of the characteristic physiological actions of mature β cells. These findings are highly significant, suggesting elevated physiological functionality of the islets generated under the angiopoietin stimulation. Furthermore, we observed more CP^+^/NKX6.1^+^ and CP^+^/MAFA^+^ cells formed in the Ang2 stimulated islets, and the insulin stimulation index in the Ang1 and Ang2 groups is comparable to that in human donor islets.

To confirm angiopoietin signaling for islet development, we examined whether Ang2 changes the glucose sensitivity of terminally differentiated insulin-secreting cells such as RIN-5F cells. RIN-5F is a rat β cell line that does not possess glucose sensitivity^45^. It secretes insulin in a non-glucose-responsive fashion. Treating RIN-5F cells with Ang2 did not result in glucose-responsive insulin secretion in these cells (Fig. S1). The results suggested that Ang2 signaling does not regulate terminally differentiated cells’ glucose-responsive insulin-secretion. Interestingly, the analysis of mRNA expression level in the iPSC-derived organoids revealed that Ang2 signaling significantly enhances not only insulin expression but also all the other major hormone gene expressions in these cells (Fig. 1d). Insulin secretion is controlled at various levels, including gene, metabolism, exocytotic, and cell–cell interaction levels^57–59^. The production of physiologically functional islets from iPSCs in vitro requires the improvement of insulin production and secretion at all levels. Our study demonstrated a critical role of angiopoietin signaling in elevating insulin gene expression, insulin-secreting cells’ F-actin remodeling, and glucose synchronized Ca^2+^ influx of islet cells.

The biological function of iPSC-derived islet cells was determined by GSIS assay. We used approximately ~ 200 islets for the assay. In order to understand how insulin secretion is affected by Ang1 and Ang2, we determined glucose synchronized Ca^2+^ influx in insulin-secreting cells at the single cell level (Fig. 4e,f). We found that most cells in the control group failed to secrete insulin efficiently upon glucose challenge. Thus, overall glucose-responsive insulin-secretion from these islets was lower, as compared to those generated under either Ang1 or Ang2 stimulation. Interestingly, we observed the heterogeneity of the glucose synchronized Ca^2+^ influx in the islets (Fig. 4f,g). This is very similar to that observed in adult donor islets. Indeed, islet heterogeneity is not unusual in adult islets^60^. There are various sub-populations of β cells with heterogeneous gene and protein expression profiles and insulin secretion capacity in adult islets^61^. Our experimental results are promising. It has been shown that the transplantation of cells capable of glucose synchronized Ca^2+^ influx can reduce hyperglycemia in mice rapidly^9^.

**Figure 8:**
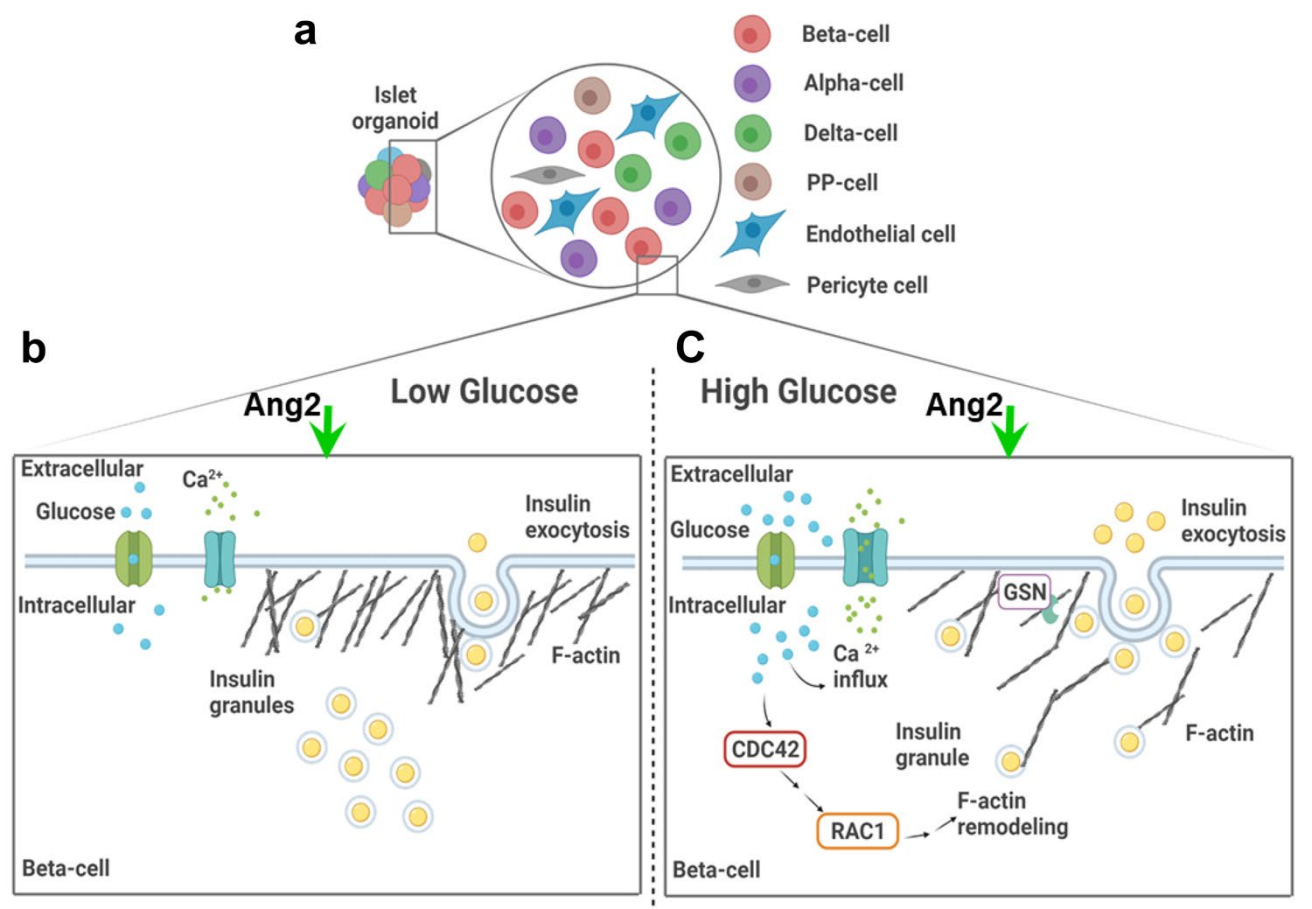
Illustration of Ang2 effect on iPSC islet differentiation and iPSC-derived islet microenvironment. (**a**) Ang2 supports islet differentiation with the generation of β, α, δ, PP-cells and additionally, promotes the generation of endothelial and pericyte cells. (**b-c**) iPSC-derived islets under Ang2 cue showed Ca^2+^ influx and regulated activity of CDC42-RAC1 pathway synchronous with glucose level change, leading to F-actin remodeling aided by gelsolin for physiologically functional insulin exocytosis. Created with BioRender.com.

The cytoskeleton of a β cell has been reported to be involved in insulin granule mobilization, fusion with the cell membrane, and finally, in facilitation of insulin exocytosis^49,62^. We observed the dynamic polymerization and depolymerization of F-actin in Ang2 stimulated islets upon glucose challenge. This observation is consistent with previous reports^63,64^. In primary human islets, the F-actin of β cells at a basal glucose condition forms a ring beneath the cell surface to block the exocytosis of insulin granules^63^. Upon glucose stimulation, cells reorganize their filaments to mobilize the insulin granules out of the cells with help of proteins like gelsolin^53,64^. Previous studies revealed that CDC42 acts as a modulator for glucose-responsive insulin secretion, which requires F-actin remodeling to mobilize granules^50,65^. Deletion of CDC42 gene in β cells decreased insulin expression and secretion in response to glucose stimulation in vitro and in vivo^66^. Moreover, RAC1 is in the downstream of CDC42 signaling^67^. The glucose-stimulated insulin secretion is decreased when RAC1 is inhibited and/or in β cell-specific RAC1 deficient mice^51^. These findings support our discovery on the unique role played by Ang2, demonstrating mechanistic insight of improved functionality of iPSC-derived islets under Ang2 stimulation for in vitro islet development as depicted in Fig. 8.

In addition, angiopoietins are known for their role in angiogenesis and vascularization^37,38^. For instance, Ang2 is involved in the maintenance of established vasculature, mainly in vascular remodeling and maturation^68^. A microvasculature in islets is a route for hormone secretion and profoundly essential for glucose and oxygen supply to execute the functions of islets. An endothelium helps induce pancreatic endocrine development^32^. In our study, we found the formation of vascular cellular components such as VE-Cadherin^+^ cells and NG2^+^ cells within the islets under the Ang2 signal. This endothelium sprouting by Ang2 might facilitate in vitro functional islet development. Taken together, the results indicated that Ang2 supports the initiation of endothelium formation along with islet differentiation which positively improves the functionality of the iPSC-derived islets.

The effect of angiopoietins has been established to be dose and context-dependent whose molecular mechanism is poorly understood^37^. Recent reports suggested their diverse roles in tissue homeostasis. In the context of tissue generation, Hu et al. stated Ang2's role in liver generation, where it controls the proliferation of hepatic cell populations in vivo^41^. Park et al. have reported the role of Ang1 in regulating insulin secretion by stabilizing the microenvironment of islets in vivo^70^. Kitazawa et al. showed that angiopoietin-like protein-2 (ANGPTL2) improves insulin sensitivity in diabetic mice^71^. In this study, we reported a previously unexplored role of angiopoietin factors played in generating mature islets from iPSCs. Our study provides new knowledge on controlling in vitro organoid development and maturation.

It is worth noting that the molecular mechanisms underlying the Ang2 effect, particularly Ang2-mediated signaling transduction pathway is unknown. More detailed characterizations are required to elucidate the mechanism of angiopoietin signaling. The studies would involve mapping Ang2 induced signaling transduction pathways using RNA-sequencing and loss- and gain-of-function assays. Unlocking of the mechanisms will help build cues critical to the production of physiologically functional islets from iPSCs for diabetes research and treatment.

## Materials and methods

### Cell culture

iPSC lines IMR90, DF4, and hESC line H9 were obtained from WiCell Research Institute. Cells were cultured on 1:100 diluted Matrigel (Corning Life Science) coated dishes and maintained in the mTeSR1 medium (StemCell Technologies), which was replenished with fresh media every day as described elsewere^71,72^. The cells were passaged every 3 to 4 days into a 1:3 split ratio using Dispase (StemCell Technologies). To initiate differentiation, the cells were seeded as single cells at one to two million cells per well of 6-well plate after Accutase (StemCell Technologies) treatment. Rat β cells RIN-5F (ATCC CRL-2058) were maintained in RPMI 1640 supplemented with 10% fetal bovine serum. Growth medium was exchanged every 3 days and cells were passaged at 80% confluence.

### Stepwise differentiation

Differentiation procedures were developed and modified based on our previous study^11^, which was initiated using optimized serum-free differentiation media with combinations of growth factors and signaling molecules (Fig. 1a). Stage I differentiation was initiated 24 h post single cell seeding. The DE cells were achieved by applying RPMI 1640 (Corning Life Science) supplemented with 50 ng/ml activin A (AA, PeproTech) and 1 mM sodium butyrate (SB, Sigma-Aldrich) for 24 h. From day 2 to day 5, the concentration of SB was reduced to 0.5 mM as described previously^73^. At Stage II, the media formulation consisted of RPMI 1640, 1 µM retinoic acid (RA, Sigma-Aldrich), 50 ng/ml keratinocyte growth factor (KGF, PeproTech), 300 nM (-)-indolactam V (ILV, AdipoGen), 100 ng/ml noggin (Nog, PeproTech), and 125 µM ascorbic acid (VitC, Sigma-Aldrich). At Stage III, cells were cultured in DMEM high glucose (Gibco) containing 1 µM RA, 300 nM ILV, 200 nM LDN 193189 (LDN, Sigma-Aldrich), 1 µM 3,3′,5-triiodo-L-thyronine Sodium Salt (T3, Sigma-Aldrich), 10 µM ALK5 inhibitor II (A5iII, Enzo Life Sciences), 100 nM γ-secretase inhibitor XX (SiXX, Millipore), and 10 µg/ml heparin (HP, Sigma-Aldrich). At Stage IV, the differentiation media was prepared with RPMI 1640 containing 1 µM T3, 10 µM A5iII, 10 µg/ml HP, 1 mM N-acetyl cysteine (NCys, Sigma-Aldrich), 0.5 µM R428 (Selleck Chem), 10 µM Trolox (Tro, Enzo Life Sciences), 100 nM SiXX, and 10 mM nicotinamide (Nic, Sigma-Aldrich); and glucose (Gibco) was supplemented up to 20 mM. Serum-free B27 (Gibco) was added from Stage I to Stage IV. At the end of Stage IV, cells were scrapped after Dispase treatment and transferred to a 24-well ultralow attachment plate (Corning Life Science) initiating Stage V. At Stage V, the differentiation media was prepared with CMRL supplement containing 2% bovine serum albumin (BSA, Sigma-Aldrich), 1 µM T3, 10 µM A5iII, 100 nM SiXX, and 10 mM Nic. The differentiation media at all stages were exchanged every two days. For suspension culture, half of the media was changed every two days. Angiopoietin proteins, Ang2 (Biolegend) and Ang1 (Peprotech) were applied to differentiation media at 20 ng/ml or indicated concentration from Stage IV until the end of Stage V.

### TaqMan quantitative real-time PCR

qRT-PCR was performed as described previously^71,72^. Briefly, RNA was extracted from cells using the RNeasy mini kit (Qiagen). Primer and probe sets targeting genes of interest were used as shown in Table S1. CFX Connect system (Bio-Rad) was used for qRT-PCR. Ct values for the target genes were normalized to internal housekeeping gene Cyclophilin A^71,73^. ΔΔCt method was employed to analyze the gene expression relative to undifferentiated cells. RNA extracted from human islets purchased from Prodo Laboratories, Inc. were used as a positive control.

### Immunostaining and confocal imaging microscopy

Islets were rinsed with Dulbecco phosphate buffered saline (DPBS) (HyClone) and fixed with 4% paraformaldehyde at room temperature for 1 to 2 h. The fixed aggregates were washed with phosphate buffered saline (PBS), and 30% sucrose (w/v) was added and stored at 4 °C overnight. Optimal Cutting Temperature compound (OCT) (ThermoFisher Scientific) was added to the samples and stored overnight at 4 °C. This was followed by snap freezing in liquid nitrogen. Thin sections, 14 µm, of the frozen samples were prepared on TruBond TM (Electron Microscopy Sciences) adhesive slides by cryosectioning. The OCT compound was removed by PBS wash. Foxp3/Transcription Factor Fixation/Permeabilization kit (ThermoFisher Scientific) was used for permeabilization, blocking and antibody staining according to the manufacturer’s instruction. Primary antibodies were applied overnight at 4 °C. Excess antibodies were washed off three times with PBS at room temperature. Secondary antibodies were then applied for 1 h at room temperature. Excess antibodies were washed off three times with PBS at room temperature. The samples were stained for nucleus with Vectashield Mounting Medium containing DAPI (ThermoFisher Scientific). Antibodies used in immunofluorescence staining were shown in Table S2. Images were captured using a Zeiss LSM 880 with an airyscan microscope. For estimating the number of different cell types, images (n = 7–16) were quantified by ImageJ (version 1.52p, https://imagej.nih.gov/ij/notes.html; or version 1.53i, https://imagej.nih.gov/ij/notes.html) using the Biovoxxel plugin v1.0 (https://www.biovoxxel.de/development/). The images were processed to remove background and noise. DAPI stained nuclei were distinguished using the watershed function. To avoid over-segmentation, the watershed function was used only when required, especially when images contained a high number of merged cells. Cell number was estimated by counting cells larger than 250-pixel units. Variations in cell size were monitored with each image and cell size threshold was modified accordingly to avoid over- or under-estimation of cell numbers.

### Glucose stimulated insulin secretion analysis

Islets were washed with DPBS and equilibrated in Krebs–Ringer buffer (KRB, Boston BioProducts) containing 1 mM glucose for 4 h. Aggregates were rinsed again with KRB containing 1 mM glucose. For p-GSIS, organoids were incubated in KRB containing 2 mM glucose and 20 mM glucose at 37 °C separately for 30 min. For multiple s-GSIS, organoids were seeded on Matrigel (1:50) coated 48-well plates and cultured in Stage V differentiation media at 37 °C overnight prior to 4 h pre-incubation step. Attached organoids were challenged serially in KRB containing (2–20 mM)–(2–20 mM)–(2–20 mM) glucose for 20 min for each stimulation condition at 37 °C. Between the three rounds of consecutive 20–2 mM challenge, the aggregates were rinsed twice by incubating in KRB containing 1 mM glucose for 5 min with each wash. The supernatants were collected from the respective glucose treatments. The number of islets under each condition was counted under an Olympus phase contrast microscope. About 100–200 µm sized aggregates were counted as clusters. Insulin was measured using an insulin ELISA kit (Mercodia) according to the manufacturer’s instructions. Insulin secretion can be normalized to single cells based on accounting of cell numbers or a measurement of DNA content^9–11^. We compared insulin secretion from the islets normalized based on organoid number or DNA content. Two normalizations gave us similar results. Thus, we presented the data based on islet number^74^. DNA of the islets was extracted using DNeasy Blood and Tissue Kit from QIAGEN following manufacturer’s instructions. Human donor islets purchased from Prodo Laboratories, Inc. (Aliso Viejo, CA) were used as positive controls. For measurement of insulin secretion from RIN-5F cells, the cells were seeded into 24-well plate at 6 × 10^4^ cells per well and allowed 72 h for complete attachment. After cell attachment, the test wells were treated with Ang2 at indicated concentration for 72 h in RPMI 1640 supplemented with 2% BSA prior to the GSIS analysis. Insulin secretion was measured using rat insulin ELISA kit (Mercodia). Cells were detached by trypsin and counted under a phase contrast microscope.

### Ca^2+^ influx imaging and data analysis

Ca^2+^ influx imaging and data analysis were performed as described elsewhere^9,75^. Briefly, islets were added on Matrigel-coated wells of 8-well chambered Labtek cover glass chamber (Nunc) and cultured for 24 h at 37 °C in the Stage V medium for attachment. They were stained with 50 µM Fluo-4 AM (ThermoFisher Scientific), a Ca^2+^ indicator, in KRB containing 1 mM glucose for 45 min at 37 °C. An additional 15 min of incubation in KRB containing 1 mM glucose was performed to rinse off the excess Ca^2+^ indicator. The aggregates were staged on an incubated stage (37 °C, 5% CO_2_) of Zeiss LSM 880 to obtain time-series images for Ca^2+^ dynamics. A sequential challenge with KRB containing (2–20 mM)–(2–20 mM)–(2–20 mM)-KCl was applied consecutively. Between the consecutive high-low (20–2 mM) glucose challenges, the aggregates were rinsed with KRB buffer containing 1 mM glucose. Fluo-4 AM intensity was recorded for 5 min for each of the incubation conditions. During the 5-min time series, a snapshot of the aggregate was captured every 17 s. For the cell-cluster level analysis, a 40 × objective was used, and for single-cell analysis, 63 ×/40 × objectives were used. Image analysis was performed with ImageJ and MATLAB (Version R2018a). A total of 126 images per aggregate, which includes 18 images per glucose treatment, were analyzed to calculate average fluorescence intensity. Individual cells of the islet organoids were also selected as region of interest (ROI) for single cell analysis. When average a.u.c at high glucose was higher than the average a.u.c at low glucose in an individual cell, the cell was considered to be responding positively. Human donor islets were used as positive controls.

### F-actin staining and image analysis

The islets generated were subjected to glucose challenges and the aggregates were fixed and stained following the immunostaining procedure mentioned above. The images were captured with Zeiss LSM 880. The β cells were identified by C-peptide staining and F-actin was visualized by Phalloidin Alexa 488 staining. Image analysis was carried out by line scan and radial profiling. To account for heterogeneity of F-actin within the β cell boundary, radial integrated intensity was assessed using ImageJ software which accounts for F-actin expression in circular of a β cell. The F-actin intensity along the center to edge represents intensities integrated around concentric circles within the ROI encompassing β cells.

### Statistical analysis

All experimental data were presented as mean ± standard deviation (SD) of at least three times of independent experiments. Two-tailed unpaired student’s t-test was used for all statistical analyses between different groups. The differences were considered to be significant when the p-value was less than 0.05. For multiple comparisons, one-way ANOVA with Turkey’s test or two-way ANOVA with Dunnett’s test was carried out.

## Supplementary Information

Supplementary Information.

## Data Availability

The raw or analyzed datasets for this study are available from the corresponding author upon reasonable request.
