# Supplementary material for: Angiopoietins stimulate pancreatic islet development from stem cells
Source: Sci Rep. 2021 Jun 30;11:13558. doi: 10.1038/s41598-021-92922-5 (PMC8245566; doi:10.1038/s41598-021-92922-5)
Supplement: Supplementary file 1 — Supplementary Information. [file 41598_2021_92922_MOESM1_ESM.docx]

**Supplementary Information**

**
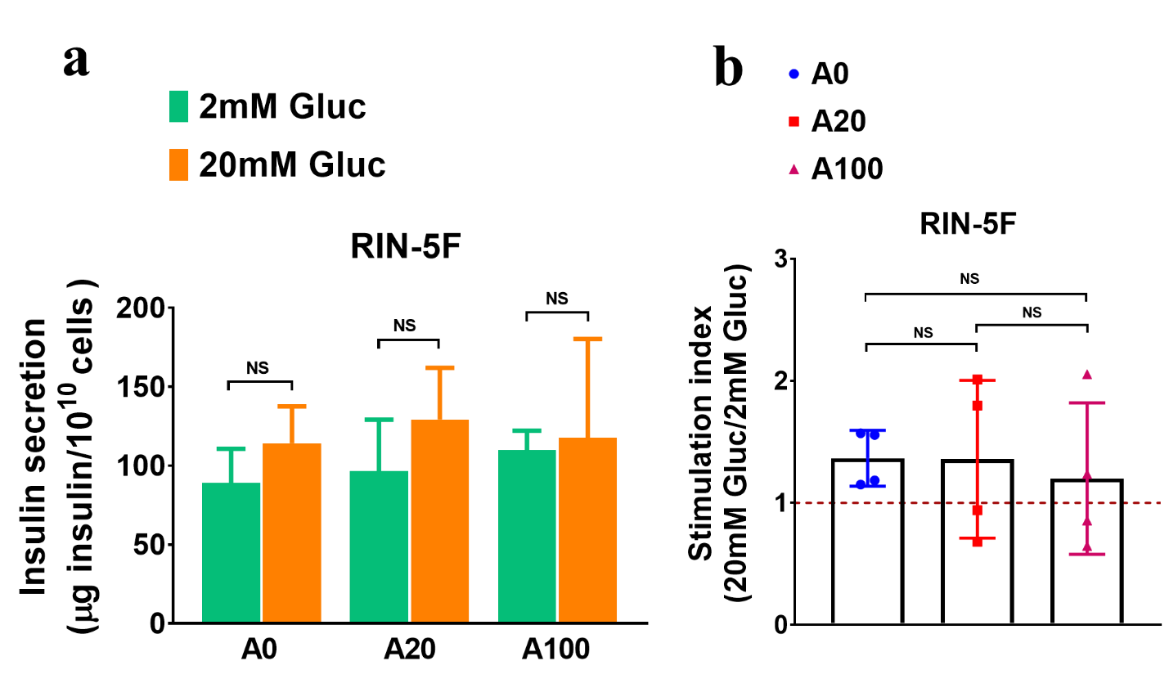
**

**Figure S1.** **The effect of Ang2 on** **glucose-stimulated insulin secretion in rat β cells.** (**a**) Parallel glucose-stimulated insulin secretion by rat β cells (n=3). RIN-5F cells were cultured in the presence of Ang2 at 20 (A20) and 100 ng/ml (A100) for 72 hours. RIN-5F cultured in the absence of Ang2 (A0) was used as control. (**b**) Stimulation index in rat β cells in the presence (A20, A100) and absence (A0) of Ang2 (n=4). Results are shown as mean ± SD. NS: not significant.

**Table S1. qRT-PCR Primers and Probes**

| Genes | Sequences of primers and probes (5’ to 3’) or Assay IDs from Applied Biosystems |
| --- | --- |
| PDX1 | Forward: CCTTTCCCATGGATGAAGTC  Reverse: CGTCCGCTTGTTCTCCTC  Probe: AAGCTCACGCGTGGAAAGGCC-BHQ |
| NKX6.1 | Hs00232355_m1 |
| MAFA | Hs01651425_s1 |
| UCN3 | Hs00846499_s1 |
| Insulin | Forward: GGGAGGCAGAGGACCTG  Reverse: CCACAATGCCACGCTTCT  Probe: FAM-AGGTGGGGCAGGTGGAGCTG-BHQ |
| Glucagon | Forward: GCTGCCAAGGAATTCATTGC  Reverse: CTTCAACAATGGCGACCTCTTC  Probe: FAM-TGAAAGGCCGAGGAAGGCGAGATT-BHQ |
| Somatostatin | Hs00356144_m1 |
| Pancreatic Polypeptide | Hs00237001_m1 |
| CDC42 | Hs00918044_g1 |
| RAC1 | Hs00251654_m1 |
| Gelsolin | Hs00609272_m1 |
| Cyclophilin A | 4310883E |

**Table S2. Antibodies and dyes used in immunofluorescence staining**

| Antibodies | Species | Manufacturer | Category | Dilution |
| --- | --- | --- | --- | --- |
| C-peptide | Rat | DSHB at University of Iowa | Primary antibody | 1:30 |
| C-peptide | Mouse | Abcam | Primary antibody | 1:200 |
| Glucagon | Rabbit | Sigma | Primary antibody | 1:50 |
| Somatostatin | Rat | Millipore | Primary antibody | 1:100 |
| Pancreatic polypeptide | Mouse | R&D SYSTEMS | Primary antibody | 1:50 |
| NKX6.1 | Mouse | DSHB at University of Iowa | Primary antibody | 1:50 |
| MAFA | Rabbit | Abcam | Primary antibody | 1:250 |
| NG2 | Rabbit | Millipore | Primary antibody | 1:100 |
| VE-Cadherin | Rabbit | Invitrogen | Primary antibody | 1:20 |
| Mouse IgG | Goat | Sigma | Secondary antibody | 1:300 |
| Rat IgG | Goat | R&D SYSTEMS | Secondary antibody | 1:60 |
| Rat IgG | Donkey | Sigma | Secondary antibody | 1:300 |
| Rabbit IgG | Goat | Sigma | Secondary antibody | 1:500 |
| Fluo-4 AM |  | Invitrogen | Dye | 1:20 |
| Phalloidin |  | Invitrogen | Dye | 1:40 |
